# Mind the context—The relevance of personality for face-to-face and computer-mediated communication

**DOI:** 10.1371/journal.pone.0272938

**Published:** 2022-08-25

**Authors:** Julian Schulze, Pavle Zagorscak, Stephen G. West, Martin Schultze, Stefan Krumm

**Affiliations:** 1 Division of Psychological Assessment, Department of Education and Psychology, Differential and Personality Psychology, Freie Universität Berlin, Berlin, Germany; 2 Division of Clinical Psychological Intervention, Department of Education and Psychology, Freie Universität Berlin, Berlin, Germany; 3 Department of Psychology, College of Liberal Arts and Sciences, Arizona State University, Tempe, AZ, United States of America; 4 Division of Psychological Methods with Interdisciplinary Focus, Institute of Psychology, Goethe-Universität, Frankfurt am Main, Germany; University of Padova, ITALY

## Abstract

A large body of research has examined the link between personality and face-to-face (FtF) communication knowledge, skills, abilities, and other characteristics (KSAOs). With the rise of digital media, text-based computer-mediated (CM) communication KSAOs have gained increasing attention. We conducted two studies to investigate how personality relates to KSAOs in the different contexts of FtF *and* CM communication. Contrasting perspectives hypothesize that the results in the FtF and CM contexts would be very similar or distinctly different. In Study 1 (*n* = 454), an online panel study, the Big Five personality dimensions were assessed and their relationships to FtF and CM communication KSAOs were investigated. Structural equation models and relative weight regression analyses showed that these personality dimensions, mostly extraversion and neuroticism, explained more variance in FtF as compared to CM communication KSAOs. Study 2 (*n* = 173), conducted in a laboratory context, showed similar results compared to Study 1. In addition, when the Big Five personality dimensions were assessed with a CM frame of reference, more variance was explained in CM than in FtF communication KSAOs. These results point to the importance of considering context effects in communication and in personality research: FtF and CM communication KSAOs need to be differentiated. If not properly contextualized, the relevance of personality and communication competencies in predicting criteria may be underestimated due to contextual mismatches.

## Introduction

With the rise of digital media, interactions are increasingly performed using text-based computer-mediated (CM) communication, for example via email or chat [1, 2]. Researchers have started to explore the knowledge, skills, abilities, and other characteristics (KSAOs) required for competent CM communication [e.g., 3]. Although some authors stress the similarity of face-to-face (FtF) and CM communication [3], others emphasize differences in the communication cues available in FtF and CM contexts (for an overview, see [4]). This controversy raises the important question about the similarities and differences between FtF and CM communication KSAOs. Is it important to consider the communication context in the assessment?

The current study adds to the understanding of the distinctiveness of FtF and CM communication KSAOs by investigating similarities and differences in their nomological network [5]. The current study includes well-established correlates of generic and oral/FtF communication competencies, i.e., personality dimensions [e.g., 6–8], and examines whether personality has similar relationships to KSAOs in FtF and CM communication. Previous research has not investigated the relationships between personality dimensions, communication KSAOs, and outcomes *from both contexts* (FtF and CM) in a single study. The current article contrasts the predictive power of the Big Five personality traits for communication KSAOs and outcomes across the FtF and CM contexts.

Investigating the distinctiveness of FtF and CM communication KSAOs is important for several reasons. If both kinds of KSAOs are distinct, contextualization may become key for successful prediction. For instance, when predicting criteria in a digital context (e.g., Facebook use, [9]), using KSAOs that are specifically contextualized to CM communication may have a predictive advantage compared to FtF communication KSAOs due to a better contextual matching (see also [10]). Training staff in the KSAOs of CM communication may be needed to acquire important 21^st^ century skills [11, 12]. In contrast, if it turned out that FtF and CM communication KSAOs cannot be differentiated, there would be no need to train staff in specific CM communication competencies. In this case, for personnel selection (e.g., for hiring virtual team members), it would likely suffice to observe the behavior of applicants in FtF interactions to gauge their communication competencies without observing their performance in digital interactions (e.g., in virtual assessment centers; [13]).

### Similarity between FtF and CM communication

In the current study, we draw on the well-established communication KSAOs model by Spitzberg [3; see also 14, 15], originally developed in the context of FtF interpersonal communication. The model proposes that competent communication requires motivation, knowledge, and skills (i.e., attentiveness, expressiveness, coordination, and composure) on the side of the communicators [3]. These variables are important predictors of communication outcomes such as satisfaction, effectiveness, and appropriateness [3, 16]. With the advent of text-based CM communication, Spitzberg extended these model components to the CM environment:

FtF and CMC interaction are more similar than they are different. Both can be explained by the same general model components, and, in most cases, the components of this model require only minor adaptation to the particular technological features of the context [3, p. 652].

Although Spitzberg [3] emphasized the similarity of FtF and CM interactions, two different general perspectives exist on this matter [see also 17]. One perspective, classical cues-filtered-out theories [18] such as media richness theory [19] and social presence theory [20] accentuate the differences between FtF and CM communication. According to media richness theory, FtF communication allows for the use of multiple cues (e.g., body language, tone of voice), immediate feedback, and a high degree of natural language [4, 19]. Social presence theory argues that FtF communication provides a greater capacity for transmitting nonverbal communication cues, which, in turn, increases a feeling of involvement and presence of other social interactors [20, 21]. The second general perspective, including social information processing theory [21] and electronic propinquity theory [22, 23], have challenged this view, arguing that with time and increasing skills, the same interaction quality can be accomplished in FtF and CM communication. Individuals adapt their communication behavior and find ways to overcome the restrictions imposed by CM communication (e.g., by using CM specific communication cues, see [24–26]). As two examples, expressiveness can be shown by relying on emoticons or explicitly stating emotions in a written message [24]. Assertiveness can be shown by using nonstandard punctuation (e.g., multiple exclamation marks, [26]). Although this second perspective recognizes the need to adapt behavior to the medium, it accentuates the similarities between both communication modes. This controversy raises the important question about the similarity of FtF and CM communication *KSAOs* and outcomes.

Empirically, several studies have shown that KSAOs in FtF/oral and text-based CM communication can indeed be differentiated [e.g., 16, 27–29]. For instance, self-reported attentiveness (i.e., the KSAO to show empathy and interest in communication partners; [3]) correlated *r* = .38 between FtF and CM contexts in a study by Schulze et al. [16]. Self-reported satisfaction (as a communication outcome in the Spitzberg model) correlated *r* = .11 between the FtF and CM contexts. The median latent (error-free) correlation between self-reported FtF and CM communication KSAOs as reported in Schulze et al. [16] was *Mdn r* = .25 and between FtF and CM communication outcomes *Mdn r* = .43 (for peer-reports: *Mdn r* = .59 and *Mdn r* = .60). These correlations can be interpreted as moderate to large (based on effect size conventions by Cohen [30]). Following the assumption that FtF and CM communication are more similar than different, higher correlations would be expected (e.g., >.80; [31]). Thus, these results suggest that communication KSAOs and outcomes have distinct components, so that that CM and FtF communication KSAOs should also be differentially predicted by personality variables.

### Personality predictors of FtF and CM communication

According to Leung and Bond [32, p. 69], “personality traits are fundamental constructs in explaining communication behavior and communication-based perceptions”. Previous literature suggests that personality is a distal, underlying driver of real-world performance, whereas KSAOs are considered more proximal determinants of behavior [11, 33]. Personality traits are viewed as predictors of both communication KSAOs and outcomes in the current study.

The existing literature has reported relationships between several broad personality variables and generic or oral/FtF interpersonal communication KSAOs and communication outcomes [e.g., 6–8, 34, 35]. For instance, Bakx et al. [34] assessed communication KSAOs (e.g., structuring a conversation, showing empathy) and found significant and positive correlations with personality traits (including emotional stability). Overall, the general finding in this line of research is that the Big Five are related to a variety of FtF communication traits.

Far less is known about the relationship between personality and CM communication KSAOs. On one hand, Ross et al. [9] found that personality variables were not significantly related to most CM communication KSAOs in the form of motivation, knowledge, and efficacy, with CM knowledge being the exception. On the other hand, Chua and Chua [36] found meaningful associations between personality traits and many CM communication KSAOs in a structural equation model (SEM). For instance, extraversion was correlated with CM motivation, knowledge, and skills. Given that the current evidence is sparse and inconsistent, no clear conclusions can be drawn about the magnitude of the relationship between personality traits and CM communication KSAOs or CM communication outcomes. One reason for the inconsistencies between FtF and CM contexts may be that traditional (generic) personality questionnaires better represent the personality in offline contexts ([37, 38]; for a summary see [39]). For instance, when thinking about the extraversion item “is outgoing, sociable” (translated from Rammstedt & John [40], p. 197), this wording may more readily prime FtF rather than CM interactions. The so-called symmetry principle predicts higher correlations between measures when the contexts of the measures match to each other [41, 42]. Consequently, generic personality measures that implicitly contain an offline framing should better match FtF than CM communication KSAOs and outcomes. Assuming the distinctiveness of both kinds of KSAOs and outcomes, we hypothesize that:

*H1a*: *Personality traits when assessed with a traditional (generic) personality questionnaire will explain more variance in FtF compared to CM communication KSAOs*.
*H1b*: *Personality traits when assessed with a traditional (generic) personality questionnaire will explain more variance in FtF compared to CM communication outcomes*.

### Contextualizing personality assessment

To specifically address whether contextualization of personality assessment is relevant for the linkage of personality with communication KSAOs and outcomes, the contextualization of the personality assessment was manipulated in the current study. Personality researchers have long recognized that the situational contexts are important drivers of variability in behavior [43]. Research has provided evidence that personality expression in digital and offline contexts differs [44]. If generic personality questionnaires do better represent the offline personality of individuals, the predictability of CM communication may be enhanced by contextualizing the personality assessment to the CM context.

One approach for contextualizing personality assessment is to use a specific contextual frame-of-reference in personality questionnaires [45]. A frame-of-reference can be provided by adding context tags that focus on a situational domain (e.g., “at work”, “at school”; [45]) to the original personality questionnaire items (e.g., “I am talkative” vs. “I am talkative at work”). Meta-analytic evidence [10] has shown that contextualized personality traits showed higher predictive power than their untagged counterpart when the contextualization (e.g., “at work”) closely mirrored the conceptual domain of the outcome (e.g., employee performance). These findings are also in line with the principle of compatibility [46] and the symmetry principle [41, 42]. Both approaches predict that higher associations between measurements can be expected if item elements such as context and time frame are matched.

Based on this reasoning, we propose that adding CM context tags to personality questionnaire items will increase the explained variance in CM communication constructs. As a result, the predictive power of personality for communication KSAOs and outcomes should be more similar across the two contexts (FtF and CM) when using context tags that match the context of assessment of the communication constructs, thereby increasing the conceptual overlap [45]:

*H2a*: *The amount of variance explained by a traditional (generic) personality questionnaire for FtF communication KSAOs will be similar to the amount of variance explained by a CM-contextualized personality questionnaire for CM communication KSAOs*.
*H2b*: *The amount of variance explained by a traditional (generic) personality questionnaire for FtF communication outcomes will be similar to the amount of variance explained by a CM-contextualized personality questionnaire for CM communication outcomes*.

Two studies were conducted to evaluate these hypotheses. Study 1 investigated the relationship of a traditional personality questionnaire (i.e., generic) on FtF and CM communication KSAOs and outcomes (see H1a and H1b) with data obtained from an online panel. Study 2 replicated Study 1 using a different item pool in a proctored laboratory setting. Study 2 also compared the use of generic *and* CM-contextualized personality traits as predictors of FtF and CM communication KSAOs and outcomes (H2a and H2b). Data and scripts to replicate the results can be obtained from the open science framework (OSF link: https://osf.io/u3jpx/). Correlation tables can be downloaded from the same repository (based on pairwise complete observations).

## Study 1

### Method

#### Ethics statement

Both studies were carried out following the ethical guidelines of the German Psychological Society (DGPs). The research in both studies was noninvasive, did not involve any intervention, did not expose participants to physical or psychological risks, nor did it affect the privacy or personal rights of the individuals. Given these study characteristics, no approval by an institutional review board (ethics committee) was required according to the guidelines of the German Psychological Society at the time of study completion.

The terms of the studies, to which all participants consented, were described right at the beginning of the survey and before any questions were asked. Participants were informed about the purpose of the study, were assured of the anonymity of their answers, given the contact information of the investigators, and assured that their participation was completely voluntary with the right to withdraw at any point in the study. Participants were explicitly informed that they were agreeing to the aforementioned terms when they clicked on the continue button.

#### Sample characteristics

Participants were recruited via an online-panel provider. The data were collected as part of a larger survey. For the current study, we only used data from participants who were not included in a previous study [16] which focused on self- and peer-reports of communication KSAOs. In the previous study, self-report data with no matchable peer report data were not included. The unused data from these unmatched participants provided the basis for Study 1 of the current article. An initial sample of *n* = 698 respondents met this inclusion criterion. Of these, *n* = 362 completed all of the personality and communication measurement instruments of the current study (i.e., complete cases). An additional *n* = 96 respondents completed all personality items and at least one full measurement battery consisting of motivation, knowledge, skills, and outcomes for either the FtF or the CM communication context, resulting in a total of *n* = 458 potentially usable cases.

Following Meade and Craig [47], the potentially usable cases were screened for irregular or careless responding. Four cases were completely excluded as almost no variability appeared in their data. The final sample size therefore equaled *n* = 454 individuals. Two additional datasets were generated for sensitivity analyses: One dataset was comprised of completers only and the second dataset consisted of participants who were *not* flagged as potentially careless responders (based on Mahalanobis distance and longstring variables that capture the maximum number of identical consecutive responses on a page). Results from these sensitivity analyses were similar to the ones reported in the main manuscript and are summarized in the Tables 1 & 2 in S1 Appendix.

The mean age of the participants was 47.43 (*SD* = 14.04). More women (60%) than men participated in the study; two individuals indicated “other” as their gender. In the full sample, 31% indicated university degree, 19% indicated a degree from a university of applied sciences, 39% indicated secondary school completion, 11% vocational training completion, <1% indicated primary school completion as their highest educational degree.

#### Assessment instruments and procedure

Demographic variables, general media usage, personality, FtF and CM communication KSAOs and communication outcomes, and three types of (communication) apprehension were assessed (see Table 1 for an overview of the constructs). Three additional questionnaires and some single items were administered, which are not considered in the present study. Questionnaires for assessing FtF and CM communication constructs were given in randomized block order.

**Table 1:** Descriptive statistics of personality variables and communication KSAOs and outcomes.

		Study 1				Study 2			
Domain	Variables	I	*M*	*SD*	*a*	I	*M*	*SD*	*a*
**Personality (generic)**	Openness	2	3.74	0.85	.50	5	3.98	0.68	.73
	Conscientiousness	2	3.57	0.78	.43	4	3.69	0.75	.71
	Extraversion	2	3.14	0.99	.77	4	3.41	0.89	.81
	Agreeableness	2	3.11	0.73	.21	4	3.10	0.77	.64
	Neuroticism	2	2.81	0.95	.67	4	2.87	0.89	.77
**Personality (CM framing)**	Openness	-^a^	-^a^	-^a^	-^a^	5	3.29	0.74	.72
	Conscientiousness	-^a^	-^a^	-^a^	-^a^	4	3.53	0.66	.58
	Extraversion	-^a^	-^a^	-^a^	-^a^	4	3.31	0.76	.77
	Agreeableness	-^a^	-^a^	-^a^	-^a^	4	3.24	0.72	.66
	Neuroticism	-^a^	-^a^	-^a^	-^a^	4	2.31	0.76	.74
**FtF communication KSAOs**	Motivation	5	4.12	0.80	.90	3	4.30	0.82	.92
	Apprehension	24	2.54	0.85	.97	4	2.34	1.01	.89
	Knowledge	4	3.99	0.79	.87	3	3.80	0.81	.78
	Attentiveness	3	4.37	0.58	.75	3	4.45	0.55	.71
	Expressiveness	4	3.89	0.72	.81	3	3.84	0.87	.83
	Composure	6	3.80	0.73	.90	3	3.71	0.80	.80
**CM communication KSAOs**	Motivation	5	3.80	0.72	.82	3	3.32	0.83	.82
	Apprehension	18	2.57	0.76	.95	3	2.18	1.01	.90
	Knowledge	4	4.00	0.66	.80	3	3.67	0.63	.54
	Attentiveness	3	3.93	0.69	.76	3	3.91	0.71	.69
	Expressiveness	4	3.58	0.73	.72	3	3.58	0.81	.61
	Composure	6	3.71	0.63	.87	3	3.64	0.64	.70
	Writing apprehension -^c^	20	2.29	0.70	.93	4	2.62	0.94	.80
**FtF communication outcomes**	Attractiveness	4	3.51	0.76	.89	-^b^	-^b^	-^b^	-^b^
	Appropriateness	4	3.95	0.64	.75	3	4.16	0.68	.69
	Effectiveness	4	3.59	0.67	.90	3	3.65	0.64	.82
	Satisfaction	4	3.88	0.86	.93	3	3.90	0.86	.92
	Clarity	4	3.89	0.68	.89	-^b^	-^b^	-^b^	-^b^
**CM communication outcomes**	Attractiveness	4	3.21	0.65	.84	-^b^	-^b^	-^b^	-^b^
	Appropriateness	4	4.27	0.61	.78	3	4.21	0.71	.76
	Effectiveness	4	3.59	0.62	.88	3	3.54	0.67	.84
	Satisfaction	4	3.70	0.72	.87	3	3.79	0.71	.89
	Clarity	4	3.88	0.59	.84	-^b^	-^b^	-^b^	-^b^
	General media usage -^c^	5	3.26	0.98	.85	5	3.44	0.88	.81

Note.

^a^ not included in Study 1

^b^ not included in Study 2

^c^ both writing apprehension and general media usage have no FtF counterpart; FtF = face-to-face, CM = computer-mediated, KSAOs = knowledge, skills, abilities, and other characteristics, I = number of items, *M* = mean, *SD* = standard deviation, *a* = coefficient alpha.

Participants reported their age, gender, education, and their general media usage as measured by the communication competence questionnaire ([3]; five-point scale: “1 = *not at all true*” to “5 = *very true*”). A 10-item short version of the Big Five inventory was administered (BFI-10, [40]; five-point scale: “1 = *not at all true*” to “5 = *very true*”) to assess openness (coefficient α = .50), conscientiousness (α = .43), extraversion (α = .77), agreeableness (α = .21), and neuroticism (α = .67).

Communication KSAOs were assessed using items from Spitzberg [3] and included motivation (FtF: α = .90; CM: α = .82), knowledge (FtF: α = .87; CM: α = .80), attentiveness (FtF: α = .75; CM: α = .76), expressiveness (FtF: α = .81; CM: α = .72), and composure (FtF: α = .90; CM: α = .87). Questionnaire items from Spitzberg [3] were used if the item content could be contextualized to both the FtF and the CM contexts (please note that the communication skill *coordination* was not assessed). Context tags relating to the FtF or the CM context were added to each item, mirroring approaches used in prior research [e.g., [37, 38]. Three different kinds of apprehensions were assessed: FtF (α = .97) and CM (α = .95) communication apprehension (using the PRCA-24 [48]; six items about oral presentation apprehension could not be translated to the CM communication questionnaire) and writing apprehension (i.e., anxiety about the writing process itself, e.g., writing an essay; α = .93; six items were omitted because they are focused on classroom writing, [49], p. 246). A five-point scale ranging from “1 = *not at all true*” to “5 = *very true*” was used for items adapted from Spitzberg [3] and McCroskey et al. [48]. Items from the Daly and Miller instrument [49] were rated from “1 = *strongly disagree*” to “5 = *strongly agree*”.

Communication outcomes were also assessed according to Spitzberg [3]. Specifically, perceived appropriateness (FtF: α = .75; CM: α = .78), effectiveness (FtF: α = .90; CM: α = .88), satisfaction (FtF: α = .93; CM: α = .87), attractiveness (FtF: α = .89; CM: α = .84), and clarity (FtF: α = .89; CM: α = .84) of communication in FtF and CM contexts were measured. A five-point scale ranging from “1 = *not at all true*” to “5 = *very true*” was used.

#### Analytic strategy

A multistep procedure was used to investigate H1a and H1b. First, SEMs using manifest variables [50] were conducted to estimate the variance explained (*R*^2^) in communication KSAOs and outcomes using the Big Five traits as predictor variables. Four separate models were estimated: Two of the models focused on the generic Big Five traits as predictors of FtF and CM communication KSAOs (H1a). These models separately estimated the proportion of variance explained in motivation, apprehension, knowledge, attentiveness, expressiveness, and composure in either the FtF or the CM condition. Additionally, the writing apprehension measure was added to both the FtF and CM models as a criterion. The other two models included the Big Five traits as predictors of the FtF and CM communication outcomes (H1b). These models separately estimated the proportion of variance in attractiveness, appropriateness, effectiveness, satisfaction, and clarity in either the FtF or the CM condition. The general media usage construct was added as a criterion in both the FtF and CM models. Based on these models, the *R*^*2*^ in each endogenous variable was compared across contexts to investigate H1a and H1b. The R-Statistics program (version 3.6.3; [51]) and the R-package lavaan (version 0.6.6; [52]) were utilized to fit the models. Full-information-maximum-likelihood estimation (FIML; [53]) was used to include individuals to address partially missing questionnaire responses in the analyses. The robust maximum likelihood estimator (MLR) was chosen to address non-normality in the data [54].

Relative weight regression analysis was used as an additional method to test the hypotheses and to learn more about the most influential personality variables [55]. Because the criterion variables were correlated with each other, multivariate relative weight analysis was used [56]. Mirroring the SEMs, four multivariate relative weight analyses were conducted with personality variables as predictors and the FtF and CM communication KSAOs as well as the outcomes representing the sets of criteria (excluding writing apprehension and general media usage). To test the hypotheses, explained variance in the FtF and CM communication KSAOs (H1a) and outcomes (H1b) criterion space was investigated (using *P*^*2*^_YX_, a “multivariate analog of *R*^*2*^*”*; see [57], p. 215). Afterwards, the significance of the weights was tested using bias corrected accelerated bootstrapped confidence intervals (*k* = 50,000 replications; alpha = .05; [55]). Each bootstrapping analysis was repeated four times. If a confidence interval consistently included or did not include zero over the four iterations, the weight was deemed non-significant (or significant). If there was inconsistency in the results across the iterations, the weight was deemed non-significant. We refer the interested reader to the (Text 1, Tables 3 & 4 in S1 Appendix) for additional details on *univariate* relative weight analyses that give further insights into the variability of relative effect sizes. R code from the RWA Web tool was used to conduct the analyses [57].

### Results

Descriptive statistics are given in Table 1. The median correlation between identical FtF and CM communication KSAOs was moderate with *Mdn r* = .30 (range: *r* = -.05 –.52). The median correlation between identical FtF and CM communication outcomes was also moderate with *Mdn r* = .42 (range: *r* = .09 –.57). The Big Five personality traits were relatively poor predictors of general media usage (4% explained variance), whereas they explained approximately 21% of variance in writing apprehension, a construct not specific to any interpersonal context.

#### Test of H1a

Results of the SEMs are presented in Table 2, column one. The total variance in communication KSAOs that was explained by personality traits using SEM was on average higher in the FtF than in the CM communication context. The amount of total explained variance ranged from 20 to 45% in the FtF context compared to 2 to 15% in the CM context. This result supports H1a.

**Table 2:** Results from structural equation models with personality traits (Generic and CM framing) as predictors of communication KSAOs and outcomes.

	Study 1	Study 2	
	*R^2^* explained by the Big Five personality traits (generic)	*R^2^* explained by the Big Five personality traits (generic)	*R^2^* explained by the Big Five personality traits (CM framing)
**FtF communication KSAOs**			
Motivation	.44	.44	.08
Apprehension	.45	.48	.13
Knowledge	.38	.60	.12
Attentiveness	.20	.35	.08
Expressiveness	.33	.57	.14
Composure	.37	.57	.17
**CM communication KSAOs**			
Motivation	.02	.03	.14
Apprehension	.15	.24	.48
Knowledge	.06	.29	.50
Attentiveness	.10	.18	.35
Expressiveness	.12	.23	.57
Composure	.10	.32	.59
Writing Apprehension -^b^	.20 (.21)	.32 (.32)	.30 (.29)
**FtF communication outcomes**			
Attractiveness	.39	-^a^	-^a^
Appropriateness	.12	.38	.31
Effectiveness	.30	.38	.09
Satisfaction	.40	.55	.17
Clarity	.29	-^a^	-^a^
**CM communication outcomes**			
Attractiveness	.10	-^a^	-^a^
Appropriateness	.10	.26	.37
Effectiveness	.05	.21	.29
Satisfaction	.03	.06	.33
Clarity	.08	-^a^	-^a^
General Media Usage -^b^	.04 (.04)	.15 (.15)	.21 (.20)

*Note*.

^a^ not included in Study 2

^b^ writing apprehension (KSAOs) and general media usage (outcomes) were included in both FtF and CM models. Parentheses show the results from the corresponding CM model; FtF = face-to-face, CM = computer-mediated, KSAOs = knowledge, skills, abilities, and other characteristics.

Results of the multivariate relative weights analyses are summarized in Table 3. The Big Five personality traits explained a higher amount of variance in the FtF (*P*^*2*^_YX_ = .128) compared to the CM (*P*^*2*^_YX_ = .070) communication KSAOs criterion space. This result supports H1a. Regarding specific personality traits, extraversion and neuroticism were associated with the largest multivariate relative weights in predicting the FtF KSAOs criterion space. Of interest, a significant multivariate relative weight for openness was found in predicting the CM rather than the FtF criterion space. These results partially support H1a.

**Table 3:** Multivariate relative weight analyses with personality (Generic and CM framing) as predictor of KSAOs and outcomes.

		Study 1						Study 2											
		Big Five personality traits (generic)						Big Five personality traits (generic)						Big Five personality traits (CM framing)					
Model	Results		O	C	E	A	N		O	C	E	A	N		O	C	E	A	N
***FtF communication KSAOs***	P^2^_YX_	.128						.134						.045					
	RW		.011	.010	**.052**	.007	**.047**		.010	.019	**.055**	.011	**.039**		.003	.006	.003	.015	.018
	Rescaled RW (%)		8.8	8.2	40.3	5.7	36.9		7.4	14.0	40.8	8.2	29.5		5.7	13.6	6.2	34.2	40.2
***CM communication KSAOs***	P^2^_YX_	.070						.092						.147					
	RW		**.015**	.006	**.015**	.009	**.024**		.019	.012	.025	.008	**.029**		**.024**	.018	**.048**	.008	**.050**
	Rescaled RW (%)		21.7	8.7	21.0	13.6	35.2		20.6	12.7	26.8	8.6	31.3		16.1	11.9	32.7	5.3	34.0
***FtF communication outcomes***	P^2^_YX_	.138						.205						.084					
	RW		.009	.009	**.064**	**.023**	**.032**		.005	**.048**	**.068**	**.036**	**.048**		.004	.015	.005	.034	.026
	Rescaled RW (%)		6.7	6.8	46.6	16.5	23.4		2.7	23.6	32.9	17.6	23.2		4.6	17.8	6.0	40.5	31.1
***CM communication outcomes***	P^2^_YX_	.053						.106						.180					
	RW		.009	.008	**.014**	**.012**	**.011**		.008	.025	.008	**.044**	.022		.016	.024	.035	.061	.044
	Rescaled RW (%)		16.4	15.7	25.5	22.3	20.2		7.3	23.1	7.5	41.3	20.8		9.1	13.1	19.4	34.1	24.3

*Note*. RW are multivariate relative weights and equal in sum (within rounding error) to the *P*^2^_YX_ that is a “multivariate analog of *R*^2^” [57, p. 215]; Rescaled RWs show percentage of the explained variance over the criterion space attributable to the individual personality variables (and will sum to 100% within rounding error); significant multivariate relative weights are bold and underlined; in Study 2, outcomes did not include attractiveness and clarity; due to missing data, *n* varies between *n* = 367 to 433 (Study 1) and *n* = 170 to 173 (Study 2) per analysis; FtF = face-to-face, CM = computer-mediated, KSAOs = knowledge, skills, abilities, and other characteristics, O = Openness, C = Conscientiousness, E = Extraversion, A = Agreeableness, N = Neuroticism

#### Test of H1b

The Big Five personality dimensions explained on average a higher amount of total variance in FtF communication outcomes than in CM communication outcomes. The total explained variance ranged from 12 to 30% (FtF) and from 3 to 10% (CM). Explained variance in appropriateness was only marginally different between contexts (12% FtF versus 10% CM). These results partially support H1b.

The Big Five personality traits explained more variance in the FtF (*P*^*2*^_YX_ = .138) compared to the CM (*P*^*2*^_YX_ = .053) communication outcomes criterion space. This result supports H1b. The largest multivariate relative weights were obtained for extraversion, neuroticism, and agreeableness in predicting the FtF criterion space, supporting H1b.

### Discussion

Study 1 addressed the question of whether the Big Five personality dimensions explain differential amounts of variance in FtF and CM communication KSAOs and outcomes. Such a finding would provide support for the assumption that FtF and CM communication KSAOs are distinct. With few exceptions, the Big Five personality variables explained a higher amount of variance in FtF communication KSAOs (H1a) and outcomes (H1b) compared to their CM counterparts, supporting the distinctiveness assumption. The traits of extraversion and neuroticism were most predictive of FtF communication KSAOs and outcomes.

We acknowledge the limitation that the 10-item personality questionnaire used in Study 1 did not capture the full domain of each of the Big Five dimensions. Some internal consistency coefficients were low as would be expected given the relationship between scale length and coefficient alpha [see e.g., 58]. A second potential limitation of Study 1 lies in the online assessment of the constructs, which may have provided an unwanted implicit form of contextualization. Hence, Study 2 aimed at replicating the Study 1 results in a face-to-face proctored laboratory environment. To investigate whether personality variables are less predictive of CM communication KSAOs per se or whether explained variance can be increased through contextualization of the personality assessment, Study 2 included a personality questionnaire with a CM frame-of-reference.

## Study 2

### Method

#### Sample characteristics

Individuals participated on a computer in a laboratory environment. From the *n* = 183 potential cases, *n* = 180 individuals provided complete data for all generic personality items and additionally one (FtF or CM) full communication motivation, knowledge, skills and outcome assessment.

Two instructed response items (e.g., *please respond to this item with “not at all true”*) and one question asking about the validity of responses were included [47]. When asked, seven individuals stated that their data were not valid and were excluded. Thus, the final sample size was *n* = 173 individuals. A dataset for sensitivity analysis was constructed consisting only of individuals who followed both instructed response items appropriately. Results from sensitivity analyses were similar to the ones reported in the main manuscript (SEMs) and are summarized in the Tables 6 & 7 in S1 Appendix.

The mean age of the full sample was 35.83 (*SD* = 16.76). Again, more women (53%) than men participated; one individual indicated “other” as gender. With respect to education, 39% of the full sample indicated completion of a university degree, 7% completed a degree from a university of applied sciences, 8% completed vocational training, 42% reported completion of secondary school, 2% reported completion of primary school, and 2% reported that they had not completed an educational degree.

#### Assessment instruments and procedure

With few exceptions, the assessment instruments closely mirrored those used in Study 1: (1) A key change from Study 1 to Study 2 was conducting analyses on the level of latent variables [50, 59]. Accordingly, to enable the specification of SEMs with well-defined measurement models in Study 2, the communication KSAOs and outcomes item pool administered in Study 1 was pruned with ant colony optimization as implemented in the R-package “stuart” (version 0.6.1; [60]). Details regarding the scale shortening are presented in the (Text 2 in S1 Appendix); (2) communication outcomes attractiveness and clarity were deleted from the questionnaire to reduce respondent burden; (3) the Big Five dimensions were assessed using the 21-item BFI-K [61] which was expected to increase reliability and breadth of content; (4) a personality questionnaire with a CM frame-of-reference was included. The administration of the inventories was similar to Study 1. The CM contextualized personality questionnaire was included at the end of the assessment.

Detailed reliability information for all Study 2 variables is summarized in Table 1. Generic personality variables were more internally consistent than in Study 1 (α range = .64 - .81). Reliabilities for the CM personality variables ranged from α = .58 - .77. The range of reliabilities for communication KSAOs was α = .71 - .92 (FtF) and α = .61 - .90 (CM), respectively. The reliabilities for the communication outcomes ranged from α = .69 - .92 (FtF) and α = .76 - .89 (CM). Finally, the reliability for media usage was α = .81 and for writing apprehension α = .80.

#### Contextualization of the personality questionnaire

The context tag “in written exchanges via digital media” was added to the item-stem of the BFI-K items measuring the Big Five. To cite one example, the item “I tend to criticize others” was reformulated to “In written exchanges via digital media I tend to criticize others”. Items with context tags were inspected and pre-tested by three judges. The three judges reached consensus that the above-described tag best maintained the meaning of the original items in the CM context.

#### Analytic strategy

First, confirmatory factor analyses (CFA) were conducted to examine if the measurement structure of the new item pool closely paralleled that of Study 1 (see Text 3 and Table 5 in S1 Appendix) for these analyses; results showed acceptable fit for the measurement models). Thereafter, the analysis strategy closely followed the strategy used in Study 1. A total of eight SEMs were specified. The first four SEMs mirrored the structure of the models tested in Study 1. This time, personality variables were included as composite scores corrected for unreliability using coefficient alpha [see 59] and communication constructs were specified as latent variables. The explained variance in the dependent variables was compared to provide a second test of H1a and H1b.

Four additional SEMs were specified using the CM personality variables as predictors of the same communication criteria, paralleling the models with generic personality variables as predictors. The explained variance in the dependent variables was inspected and compared to probe H2a and H2b. Considering the large model complexity and the more limited sample size, sensitivity analyses were conducted with smaller df models by treating each criterion (FtF/CM) as a single outcome (see Tables 8 & 9 in S1 Appendix for these analyses). All models were considered acceptable with a Comparative Fit Index (CFI) ≥.90, Root Mean Square Error of Approximation (RMSEA) ≤.08, and Standardized Root Mean Square Residual (SRMR) ≤.10 [62, 63].

Paralleling Study 1, the multivariate relative weight analyses were computed at the manifest scale level using the generic personality variables as predictors. The comparison of *P*^*2*^_YX_ again served as a test of H1a and H1b. The procedures were repeated with the CM personality test scores as predictors and *P*^*2*^_YX_ compared to test H2a and H2b. Again, we refer the interested reader to the (Tables 10–12 in S1 Appendix) for additional details on *univariate* relative weight analyses.

### Results

Descriptive statistics are presented in Table 1. The median correlation between identical FtF and CM communication KSAOs was *Mdn r* = .25 (range: *r* = -.11 –.41). The median correlation between identical FtF and CM communication outcomes was *Mdn r* = .38 (range: *r* = .03 –.56). Except for agreeableness, the mean scores for CM personality were descriptively lower compared to the generic personality version. The correlations between generic and CM personality variables were moderate to large following Cohen’s [30] descriptive guidelines (openness: *r* = .56; conscientiousness: *r* = .43; extraversion: *r* = .37; agreeableness: *r* = .59; neuroticism: *r* = .50). All eight estimated SEMs exhibited acceptable fit (lowest robust CFI = .91; highest robust RMSEA = .06; highest SRMR = .06; see Table 5 in S1 Appendix for details). The CM Big Five personality traits explained more variance in general media usage than the generic Big Five (21% versus 15%), whereas they explained approximately the same amount of variance in writing apprehension.

#### Second set of tests of H1a and H1b

In the following, the findings of the second set of tests of H1a and H1b are briefly summarized. As can be seen from Table 2 (column 2), generic personality variables explained more variance in FtF compared to CM communication KSAOs and outcomes in each case. The multivariate relative weights analyses showed that generic personality variables again explained more variance in the FtF (*P*^*2*^_YX_ = .134) than in the CM (*P*^*2*^_YX_ = .092) communication KSAOs criterion space (see Table 3). The same was true for the communication outcomes criterion space (FtF *P*^*2*^_YX_ = .205 versus CM *P*^*2*^_YX_ = .106). Some of the multivariate relative weights that were significant in Study 1 did not reach statistical significance over all 4 bootstrap iterations in Study 2. This finding may partially be a result of the differential criterion space (communication outcomes clarity and attractiveness that were included in Study 1 were not assessed) and the smaller sample size. In sum, these results largely speak to successful replication of most of the major Study 1 findings.

#### Tests of H2a

The explained variance in CM communication KSAOs increased when using CM personality variables as predictors (see Table 2, column 3). However, the proportion of variance explained did not reach the level attained by generic personality variables predicting FtF communication KSAOs in each case. For example, the explained variance in CM motivation (14%) using CM personality variables as predictors did not reach the levels attained for FtF motivation (44%) using the generic personality variables as the predictors. This finding partially supports H2a –the amount of variance explained was not completely balanced for each communication KSAO.

When using CM framed personality variables as predictors of CM communication KSAOs in the multivariate analysis, the *P*^2^_YX_ for the KSAOs was similar in magnitude compared to using generic personality variables as predictors of FtF communication KSAOs (*P*^2^_YX_ = .147 versus *P*^2^_YX_ = .134). This finding supports H2a. CM extraversion and CM neuroticism had the largest weights, followed by CM openness.

#### Tests of H2b

The explained variance in CM communication outcomes again increased with CM personality variables as predictors. Similar to the KSAOs, however, the variance explained in communication outcomes was not completely balanced for each communication outcome. For instance, the explained variance in CM satisfaction (33%) using CM personality variables as predictors did not reach the levels attained for FtF satisfaction (55%) using the generic personality variables as the predictors. Thus, the findings partially support H2b.

CM framed personality was somewhat less predictive of the CM outcome criterion space compared to generic personality traits predicting the FtF outcome criterion space, (*P*^2^_YX_ = .180 versus *P*^2^_YX_ = .205). Although the weights of CM personality variables in predicting the CM criterion space were of moderate magnitude, they did not differ from 0 in each of the four bootstrap iterations. Some of the reported results may be the consequence of the smaller sample size of Study 2. Thus, these results only partially support H2b.

### Discussion

Study 2 findings replicated most of the Study 1 results in a proctored laboratory environment. Furthermore, the results were generally supportive of the idea that adding a CM frame-of-reference to personality questionnaires would increase the amount of explained variance for the CM communication KSAOs and communication outcomes. However, the variance accounted for by CM contextualized personality traits did not reach the same level as their generic counterparts in predicting FtF communication constructs in each case, only partially supporting H2a and H2b.

## General discussion

Given the rapid expansion of text-based CM communication [1], understanding similarities and differences between FtF and CM communication competencies is an important topic. Reflecting the clear distinction between two perspectives on the similarity of FtF and CM communication channels [e.g., 3, 4], this article addressed the question of whether CM communication KSAOs should be treated as distinct from traditional FtF communication KSAOs. The current study used the Big Five personality traits as predictor variables of FtF and CM communication KSAOs and outcomes to probe this question.

### Theoretical contributions

The first important conclusion that can be drawn from the results of the two studies is that personality variables derived from generic personality inventories are important predictors of FtF communication KSAOs, but less so of CM communication KSAOs. The contextualized communication KSAOs share only a moderate amount of variance with each other, which is in line with prior research [16, 27–29]. They are also predicted differentially by the Big Five personality variables. This result highlights that CM and FtF communication KSAOs have distinct components. Researchers need to acknowledge the contextual features of their studies and tailor their communication measures to the contexts under investigation. For instance, if criteria in digital contexts such as social media use need to be predicted [9], CM specific communication KSAOs are contextually more symmetric with the criterion which in turn should increase their predictive power [41, 42].

A second contribution is that, if properly contextualized, the Big Five personality variables can be important predictors of CM communication KSAOs. In accordance with prior research [37], personality scores differed between the generic and the CM specific assessment of Big Five traits. There was considerable non-shared variance between identical Big Five traits when correlating the two versions, indicating that individuals saw limited personality consistency from one context to the other. The variance explained in CM communication KSAOs and outcomes by the CM specific personality traits in Study 2 was higher than the explained variance by their generic counterparts. This finding is in line with recent developments in research on the relationship of personality and work performance. Shaffer and Postlethwaite [10] found personality variables contextualized to the work context showed higher predictive power for work performance than their untagged counterparts due to a higher conceptual overlap. Generic personality measures may better represent the FtF than the online behavior of an individual ([37, 38]; but see the limitations section), making them more symmetric to FtF communication criteria. Thus, matching the context in the assessment of communication *and* in the assessment of personality seems of utmost importance.

Our third contribution is the identification of the most important individual predictors of communication KSAOs and outcomes as conceptualized in the communication competence framework by Spitzberg [3] using relative weight analyses. The current study underscores the importance of extraversion and neuroticism as predictors of communication KSAOs and outcomes. Previous studies [e.g., 6, 35] have found these variables to be of importance in the context of communication. Our design went beyond previous research, allowing to explore the importance of these personality variables across two communication contexts embedded in the well-established KSAOs model [3, 14] using both generic *and* CM contextualized personality variables as predictors. Our results point to their cross-contextual relevance, which is a novel finding.

### Practical contributions

The current findings support prior arguments that CM communication competencies may be important 21^st^ century skills [11, 12]. In personnel selection processes, assessors may benefit from also considering CM communication KSAOs in addition to classical FtF communication competencies. For instance, in addition to FtF communication exercises in assessment centers (e.g., FtF group discussions [64]), assessors could also observe the behavior of applicants in digital communication contexts within a virtual assessment center [13]. By doing this, assessors would gain a better impression of the specific CM communication competencies of job applicants that potentially differ from their FtF communication competencies. Personnel development programs may profit from training specifically focused on improvement of CM communication KSAOs. For instance, individuals could be trained in the use of nonverbal cues such as emoticons [24], nonstandard punctuation [26], and chronemics (e.g., response latencies in CM interactions, [25]).

### Limitations and future research

There are several important limitations of the present work that should be addressed in future research. First, this study was based on self-reported *perceived* communication KSAOs and outcomes. This approach is limited in two ways: (1) The use of a common method might have influenced the results found in this study. We attempted to minimize method bias by providing evidence of construct validity (factorial validity of the measures, indications of convergent and discriminant validity for the measures, see [65]). However, extending the study design to include other methods besides self-report would more directly address this issue. (2) *Perceived* communication competence has been criticized for not being an accurate indicator of actual competence [66]. In the present study the focus was on perceptions of FtF and CM communication KSAOs and outcomes as well as on their relationship with personality variables rather than on their association with actual communication performance. Convergence of the findings with more objective indicators of communication KSAOs should be investigated (e.g., chat protocols, FtF video assessments).

Second, although the item tag for the CM context was carefully constructed, it cannot be ruled out that not every adapted personality item functioned in an optimal manner. Some items might be prone to difficulties in interpretation. Our preliminary item metric analysis did not identify problems and the debriefing interviews with participants in Study 2 did not elicit negative feedback about the items. Nonetheless, additional scale development work is needed. Systematized thinking aloud techniques could be used to explore the thoughts associated with CM contextualized items in more depth (see [67], for a methods overview).

Third, although Study 2 incorporated a longer 21-item Big Five personality inventory than the one used in Study 1, this instrument still lacks the content breadth inherent in more comprehensive personality questionnaires. The popular hierarchical NEO framework by Costa and McCrae [68] consists of 6 facets underlying each broad trait leading to a total of 240 items (NEO-PI-R). A more detailed analysis of the broader and more specific aspects of personality that are important for the prediction of communication KSAOs would be of value.

Fourth, a personality inventory with an explicit FtF framing in Study 2 was not assessed. Comparing correlations between the generic personality inventory and a CM version on the one hand and between the generic personality inventory and a FtF version on the other hand may be useful to provide additional evidence for a “hidden” offline frame-of-reference in generic personality questionnaires [see 41].

Finally, other aspects of the context can influence perceptions of communication competence besides the communication medium such as the communicators’ relationship [3]. For example, it would be interesting to learn the degree to which CM expressivity with coworkers converges with CM expressivity with managers. Similarly, in the current study, e-mail, chat, and forums were all subsumed under the umbrella term “text-based CM communication”. Yet, these media devices differ in several aspects, including their transmission and processing capabilities [69]. Future studies of convergence of the KSAOs should be examined across more diverse CM contexts.

## Conclusion

Two studies were presented in which the Big Five personality traits were used to predict FtF and CM communication criteria. This was done to investigate if CM communication KSAOs should be conceptualized as distinct from their classical FtF counterpart. Personality traits assessed using a traditional (generic) personality inventory were important predictors of FtF but were much less predictive of CM communication KSAOs. In contrast, a CM contextualized assessment of the same personality traits showed the opposite prediction pattern. Our results highlight the importance of considering context effects in communication and personality research alike.

## Supporting information

S1 Appendix Appendix with supporting information for the main manuscript. (DOCX)
