# Supplementary material for: Mind the context—The relevance of personality for face-to-face and computer-mediated communication
Source: PLoS One. 2022 Aug 25;17(8):e0272938. doi: 10.1371/journal.pone.0272938 (PMC9409523; doi:10.1371/journal.pone.0272938)
Supplement: S1 Appendix — (DOCX) [file pone.0272938.s001.docx]

Supplemental material for the article

Mind the Context – The Relevance of Personality for Face-to-face and Computer-Mediated Communication

**Table of Contents**

[**Supplemental material for Study 1** 3](#_Toc108701484)

[**Table 1** 4](#_Toc108701485)

[**Table 2** 5](#_Toc108701486)

[**Text 1: Univariate Relative Weights Analyses** 6](#_Toc108701487)

[**Table 3** 7](#_Toc108701488)

[**Table 4** 8](#_Toc108701489)

[**Text 2: Ant Colony Optimization Results** 9](#_Toc108701490)

[**Supplemental material for Study 2** 11](#_Toc108701491)

[**Text 3: Test of Measurement Models** 12](#_Toc108701492)

[**Table 5** 13](#_Toc108701493)

[**Table 6** 14](#_Toc108701494)

[**Table 7** 15](#_Toc108701495)

[**Table 8** 16](#_Toc108701496)

[**Table 9** 17](#_Toc108701497)

[**Table 10** 18](#_Toc108701498)

[**Table 11** 19](#_Toc108701499)

[**Table 12** 20](#_Toc108701500)

[**References** 21](#_Toc108701501)

# **Supplemental material for Study 1**

## **Table 1**

*Sensitivity analysis (completers only, n = 359). Personality variables as predictors of communication KSAOs and Outcomes*

|  | **Study 1** |  |
| --- | --- | --- |
|  | ***R²* explained by the Big Five personality traits (generic)** |  |
| **FtF communication KSAOs** |  |  |
| Motivation | .46 |  |
| Apprehension | .46 |  |
| Knowledge | .42 |  |
| Attentiveness | .20 |  |
| Expressiveness | .35 |  |
| Composure | .38 |  |
| **CM communication KSAOs** |  |  |
| Motivation | .02 |  |
| Apprehension | .15 |  |
| Knowledge | .05 |  |
| Attentiveness | .09 |  |
| Expressiveness | .10 |  |
| Composure | .08 |  |
| Writing Apprehension -^a^ | .19 (.19) |  |
| **FtF communication outcomes** |  |  |
| Attractiveness | .40 |  |
| Appropriateness | .15 |  |
| Effectiveness | .31 |  |
| Satisfaction | .43 |  |
| Clarity | .33 |  |
| **CM communication outcomes** |  |  |
| Attractiveness | .10 |  |
| Appropriateness | .12 |  |
| Effectiveness | .04 |  |
| Satisfaction | .02 |  |
| Clarity | .07 |  |
| General Media Usage -^a^ | .08 (.08) |  |

*Note.* ^a^ writing apprehension (KSAOs) and general media usage (outcomes) were included in both FtF and CM models. Parentheses show the results from the corresponding CM model; FtF = face-to-face, CM = computer-mediated, KSAOs = knowledge, skills, abilities, and other characteristics

## **Table 2**

*Sensitivity analysis (potential careless responders excluded, n = 393). Personality variables as predictors of communication KSAOs and Outcomes*

|  | **Study 1** |  |
| --- | --- | --- |
|  | ***R²* explained by the Big Five personality traits (generic)** |  |
| **FtF communication KSAOs** |  |  |
| Motivation | .44 |  |
| Apprehension | .43 |  |
| Knowledge | .38 |  |
| Attentiveness | .21 |  |
| Expressiveness | .33 |  |
| Composure | .35 |  |
| **CM communication KSAOs** |  |  |
| Motivation | .03 |  |
| Apprehension | .18 |  |
| Knowledge | .07 |  |
| Attentiveness | .13 |  |
| Expressiveness | .13 |  |
| Composure | .11 |  |
| Writing Apprehension -^b^ | .23 (.24) |  |
| **FtF communication outcomes** |  |  |
| Attractiveness | .39 |  |
| Appropriateness | .11 |  |
| Effectiveness | .32 |  |
| Satisfaction | .41 |  |
| Clarity | .28 |  |
| **CM communication outcomes** |  |  |
| Attractiveness | .11 |  |
| Appropriateness | .11 |  |
| Effectiveness | .06 |  |
| Satisfaction | .04 |  |
| Clarity | .07 |  |
| General Media Usage -^b^ | .04 (.04) |  |

*Note.* ^a^ writing apprehension (KSAOs) and general media usage (outcomes) were included in both FtF and CM models. Parentheses show the results from the corresponding CM model; FtF = face-to-face, CM = computer-mediated, KSAOs = knowledge, skills, abilities, and other characteristics

## **Text 1: Univariate Relative Weights Analyses**

This section reports additional analyses focusing on the relative effect sizes of personality variables. Specifically, the multivariate relative weight analyses were followed up by univariate relative weight analyses (Tonidandel et al., 2009), in which we treated each FtF and CM construct as a single criterion. This approach was taken to identify potential variability in the relative effect sizes of the personality variables. In addition to the individual results, the overall pattern of results was inspected by averaging the relative weights across traits within each communication context, separated by KSAOs and outcomes. Furthermore, the median and the range of the univariate raw relative weights were computed (see supplementals for detailed results). We used R code from the RWA Web tool (Tonidandel & LeBreton, 2015). The results of the univariate relative weights analyses can be obtained from Supplemental Table 3 & 4 (Study 1). Study 2 re-investigated the Study 1 findings and additionally considered CM personality variables as predictors. The results for the Study 2 univariate relative weights analyses can be obtained from Supplemental Table 8, 9, and 10.

## **Table 3**

*Univariate relative weight analyses: Individual results for multiple regression models (generic personality as predictor; full sample)*

| Univariate relative  weight analysis | |  |  | **Predictor Variables - Big Five generic** | | | | |
| --- | --- | --- | --- | --- | --- | --- | --- | --- |
|  |  |  |  | **Relative weights** | | | | |
|  |  | *n* | Model *R^2^* | O | C | E | A | N |
| **KSAOs** | |  |  |  |  |  |  |  |
| Motivation FtF | | 428 | 0.44 | 0.01 | 0.03 | 0.26 | 0.01 | 0.13 |
| Motivation CM | | 434 | 0.02 | 0.00 | 0.00 | 0.01 | 0.00 | 0.00 |
| Apprehension FtF | | 367 | 0.46 | 0.01 | 0.01 | 0.16 | 0.00 | 0.27 |
| Apprehension CM | | 368 | 0.15 | 0.01 | 0.00 | 0.04 | 0.00 | 0.08 |
| Knowledge FtF | | 428 | 0.38 | 0.01 | 0.03 | 0.20 | 0.00 | 0.14 |
| Knowledge CM | | 434 | 0.06 | 0.04 | 0.01 | 0.00 | 0.00 | 0.01 |
| Attentiveness FtF | | 428 | 0.19 | 0.02 | 0.02 | 0.09 | 0.01 | 0.05 |
| Attentiveness CM | | 434 | 0.10 | 0.04 | 0.03 | 0.02 | 0.01 | 0.00 |
| Expressiveness FtF | | 428 | 0.33 | 0.05 | 0.01 | 0.21 | 0.00 | 0.06 |
| Expressiveness CM | | 433 | 0.12 | 0.07 | 0.00 | 0.04 | 0.01 | 0.00 |
| Composure FtF | | 428 | 0.36 | 0.01 | 0.02 | 0.17 | 0.00 | 0.15 |
| Composure CM | | 433 | 0.10 | 0.04 | 0.01 | 0.01 | 0.01 | 0.03 |
|  |  |  |  |  |  |  |  |  |
|  |  | *n* | Model *R^2^* | O | C | E | A | N |
| **Outcomes** |  |  |  |  |  |  |  |  |
| Attractiveness FtF | | 428 | 0.39 | 0.02 | 0.01 | 0.24 | 0.04 | 0.09 |
| Attractiveness CM | | 433 | 0.09 | 0.02 | 0.01 | 0.06 | 0.01 | 0.00 |
| Appropriateness FtF | | 428 | 0.12 | 0.03 | 0.00 | 0.00 | 0.07 | 0.02 |
| Appropriateness CM | | 433 | 0.10 | 0.02 | 0.02 | 0.00 | 0.04 | 0.02 |
| Effectiveness FtF | | 428 | 0.29 | 0.02 | 0.03 | 0.13 | 0.00 | 0.12 |
| Effectiveness CM | | 433 | 0.04 | 0.02 | 0.01 | 0.01 | 0.00 | 0.00 |
| Satisfaction FtF | | 428 | 0.40 | 0.00 | 0.02 | 0.24 | 0.02 | 0.12 |
| Satisfaction CM | | 433 | 0.03 | 0.01 | 0.00 | 0.01 | 0.00 | 0.00 |
| Clarity FtF |  | 428 | 0.29 | 0.01 | 0.04 | 0.10 | 0.01 | 0.13 |
| Clarity CM |  | 433 | 0.08 | 0.01 | 0.03 | 0.01 | 0.00 | 0.03 |

*Note.* FtF = face-to-face, CM = computer-mediated, KSAOs = knowledge, skills, abilities, and other characteristics, O = Openness, C = Conscientiousness, E = Extraversion, A = Agreeableness, N = Neuroticism

## **Table 4**

*Summary of univariate relative weight analyses with personality (generic) as predictor of KSAOs and Outcomes (full sample*)

|  |  | | **Study 1** | | | | | |
| --- | --- | --- | --- | --- | --- | --- | --- | --- |
|  |  | | **Big Five personality traits  (generic)** | | | | | |
| **Model** | | **Results** | | O | C | E | A | N |
| ***FtF communication KSAOs*** | | Mean RW | | .02 | .02 | .18 | .01 | .13 |
|  | | Median RW | | .01 | .02 | .19 | .00 | .13 |
|  | | Range RW | | .01-.05 | .01-.03 | .09-.26 | .00-.01 | .05-.27 |
| ***CM communication KSAOs*** | | Mean RW | | .03 | .01 | .02 | .01 | .02 |
|  | | Median RW | | .04 | .00 | .02 | .01 | .01 |
|  | | Range RW | | .00-.07 | .00-.03 | .00-.04 | .00-.01 | .00-.08 |
| ***FtF communication outcomes*** | | Mean RW | | .01 | .02 | .14 | .03 | .10 |
|  | | Median RW | | .02 | .02 | .13 | .02 | .12 |
|  | | Range RW | | .00-.03 | .00-.04 | .00-.24 | .00-.07 | .02-.13 |
| ***CM communication outcomes*** | | Mean RW | | .02 | .01 | .02 | .01 | .01 |
|  | | Median RW | | .02 | .01 | .01 | .00 | .00 |
|  | | Range RW | | .01-.02 | .00-.03 | .00-.06 | .00-.04 | .00-.03 |

*Note:* RW are univariate relative weights; *n* varies between *n* = 367 to 434 (Study 1); FtF = face-to-

face, CM = computer-mediated, KSAOs = knowledge, skills, abilities, and other characteristics, O = Openness, C = Conscientiousness, E = Extraversion, A = Agreeableness, N = Neuroticism.

## **Text 2: Ant Colony Optimization Results**

As outlined in the main manuscript, we pruned the item pool administered in Study 1 with ant colony optimization as implemented in the R-package “stuart” (version 0.6.1; Schultze, 2017). Communication KSAOs and outcomes were pruned in accordance with the underlying theoretical frameworks (i.e., Daly & Miller, 1975; McCroskey et al., 1985; Spitzberg, 2006). The scales were pruned by jointly optimizing the values of the Comparative Fit Index (CFI), the Root Mean Square Error of Approximation (RMSEA), and the Standardized Root Mean Square Residual (SRMR) (West et al., 2012) as well as the approximation to the manifest correlations of the full item scales using maximum likelihood estimation. All models were considered acceptable with CFI ≥ .90, RMSEA ≤ .08, and SRMR ≤ .10 (Schweizer, 2010; West et al., 2012).

To construct well defined measurement models, three separate models were estimated and pruned with the help of ant colony optimization. One model included motivation, knowledge, and the three skills in both the FtF and CM contexts (10-factor model); one model included the three types of apprehensions (3-factor model); and one model was comprised of the five communication outcome variables in both the FtF and CM contexts (10-factor model). The five items of the general media usage scale were neither pruned nor included in the models. Importantly, each communication context was treated as a method in the pruning algorithm to ensure that the final pruned scales were comprised of the exact same items across both contexts. One exception to this rule was the construct of FtF and CM communication apprehension. Here, the pruned FtF communication apprehension scale comprised of 1 item more than the CM counterpart to capture the public speaking content that was not transferable to the CM domain. The pruning process resulted in communication KSAOs and outcome scales with acceptable fit statistics within each of the proposed models in Study 1. Specifically, the pruned 10-factor KSAOs model exhibited a CFI = .93, RMSEA =.06, and SRMR = .04. The pruned 3-factor apprehensions model resulted in a CFI = 0.99, RMSEA = .03, and SRMR = .03. The reduced 10-factor outcome model resulted in a CFI = .95, RMSEA = .05, and a SRMR = .04. Finally, an

additional model that also included the five items of the general media usage scale along the FtF and CM communication outcomes also fitted the data well, with CFI = .95, RMSEA = .04, and a SRMR = .04. This reduced item pool was deemed adequate for usage in Study 2.

# **Supplemental material for Study 2**

## **Text 3: Test of Measurement Models**

As outlined in the main manuscript, we tested the measurement models of the pruned item pool as constructed in Study 1 with the data obtained in Study 2. In total, three confirmatory factor analyses were estimated: One model included all communication KSAOs in FtF and CM contexts (motivation, knowledge, and the three skills in both the FtF and CM contexts), another model included the three apprehensions (FtF and CM communication apprehension as well as writing apprehension), and the last model included FtF and CM communication outcomes (FtF and CM effectiveness, appropriateness, and satisfaction as well as general media usage). FIML was used to account for partially missing data and the MLR estimator was chosen to account for non-normality in the data. Results for the three models can be obtained from Supplemental Table 4a (see next page).

## **Table 5**

*Robust fit-statistics for all CFA and SEM models (full sample; Study 2)*

|  |  | **χ²** | **df** | **χ²- p value** | **CFI** | **RMSEA** | **SRMR** |
| --- | --- | --- | --- | --- | --- | --- | --- |
| CFA Model-fit | Model for KSAOs (*n* = 173) | 551.989 | 360 | <.001 | .91 | .06 | .07 |
|  | Model for Apprehensions (*n* = 171) | 79.971 | 41 | <.001 | .96 | .08 | .07 |
|  | Model for Outcomes (*n* = 173) | 276.421 | 209 | .001 | .96 | .05 | .05 |
| SEM Model-fit | **Big Five generic as predictors** |  |  |  |  |  |  |
|  | Model for FtF KSAOs (*n* = 173) | 375.689 | 289 | <.001 | .96 | .04 | .06 |
|  | Model for CM KSAOs (*n* = 173) | 379.986 | 263* | <.001 | .92 | .05 | .06 |
|  | Model for FtF outcomes (*n* = 173) | 181.626 | 121 | <.001 | .95 | .06 | .05 |
|  | Model for CM outcomes (*n* = 173) | 178.163 | 121 | .001 | .95 | .05 | .05 |
|  | **Big Five CM contextualized as predictors** |  |  |  |  |  |  |
|  | Model for FtF KSAOs (*n* = 172) | 372.947 | 289 | <.001 | .96 | .04 | .05 |
|  | Model for CM KSAOs (*n* = 173) | 421.913 | 263* | <.001 | .91 | .06 | .06 |
|  | Model for FtF outcomes (*n* = 173) | 157.560 | 121 | <.001 | .97 | .04 | .05 |
|  | Model for CM outcomes (*n* = 173) | 178.492 | 121 | .001 | .95 | .05 | .05 |

*Note.* * the df for the CM KSAOs models differ from the FtF KSAOs models: The FtF apprehension scale consists of one more item

(i.e., oral communication apprehension item; see main text); FtF = face-to-face, CM = computer-mediated, KSAOs = knowledge, skills,

abilities, and other characteristics

## **Table 6**

*Robust fit-statistics for all CFA and SEM models (potential careless responders excluded; Study 2)*

|  |  | **χ²** | **df** | **χ²-**  **p value** | **CFI** | **RMSEA** | **SRMR** |
| --- | --- | --- | --- | --- | --- | --- | --- |
|  |  |  |  |  |  |  |  |
| CFA Model-fit | Model for KSAOs (*n* = 124) | 530.069 | 360 | <.001 | .90 | .06 | .08 |
|  | Model for Apprehensions (*n* = 124) | 65.571 | 41 | .009 | .97 | .07 | .07 |
|  | Model for Outcomes (*n* = 124) | 282.168 | 209 | .001 | .95 | .05 | .07 |
| SEM Model-fit | **Big Five generic as predictors** |  |  |  |  |  |  |
|  | Model for FtF KSAOs (*n* = 124) | 357.904 | 289 | .004 | .96 | .04 | .06 |
|  | Model for CM KSAOs (*n* = 124) | 363.864 | 263* | <.001 | .92 | .06 | .07 |
|  | Model for FtF outcomes (*n* = 124) | 167.010 | 121 | .004 | .95 | .06 | .06 |
|  | Model for CM outcomes (*n* = 124) | 166.708 | 121 | .004 | .95 | .06 | .07 |
|  | **Big Five CM contextualized as predictors** |  |  |  |  |  |  |
|  | Model for FtF KSAOs (*n* = 123) | 340.760 | 289 | .020 | .97 | .04 | .06 |
|  | Model for CM KSAOs (*n* = 123) | 394.858 | 263* | <.001 | .90 | .06 | .08 |
|  | Model for FtF outcomes (*n* = 123) | 152.781 | 121 | .027 | .96 | .05 | .06 |
|  | Model for CM outcomes (*n* = 123) | 188.616 | 121 | .001 | .93 | .07 | .08 |

*Note.* * the df for the CM KSAOs models differ from the FtF KSAOs models: The FtF apprehension scale consists of one more item

(i.e., oral communication apprehension item; see main text); to reduce model complexity, we fitted all models without FIML and the associated mean structure. Therefore, one case is missing in the CM contextualized personality condition; FtF = face-to-face, CM = computer-mediated, KSAOs = knowledge, skills, abilities, and other characteristics

## **Table 7**

*Sensitivity analysis (potential careless responders excluded). Personality variables as predictors of communication KSAOs and Outcomes*

|  |  | **Study 2** | |
| --- | --- | --- | --- |
|  |  | ***R²* explained by the Big Five personality traits (Generic *n =* 124)** | ***R²* explained by the Big Five personality traits (CM framing *n =* 123)** |
| **FtF communication KSAOs** |  |  |  |
| Motivation |  | .47 | .07 |
| Apprehension |  | .49 | .08 |
| Knowledge |  | .54 | .07 |
| Attentiveness |  | .33 | .09 |
| Expressiveness |  | .66 | .08 |
| Composure |  | .62 | .13 |
| **CM communication KSAOs** |  |  |  |
| Motivation |  | .05 | .13 |
| Apprehension |  | .15 | .41 |
| Knowledge |  | .43 | .47 |
| Attentiveness |  | .12 | .39 |
| Expressiveness |  | .23 | .54 |
| Composure |  | .23 | .54 |
| Writing Apprehension -^b^ |  | .35 (.35) | .25 (.25) |
| **FtF communication outcomes** |  |  |  |
| Attractiveness |  | -^a^ | -^a^ |
| Appropriateness |  | .27 | .22 |
| Effectiveness |  | .36 | .11 |
| Satisfaction |  | .54 | .15 |
| Clarity |  | -^a^ | -^a^ |
| **CM communication outcomes** |  |  |  |
| Attractiveness |  | -^a^ | -^a^ |
| Appropriateness |  | .24 | .47 |
| Effectiveness |  | .20 | .32 |
| Satisfaction |  | .05 | .35 |
| Clarity |  | -^a^ | -^a^ |
| General Media Usage -^b^ |  | .20 (.20) | .16 (.16) |

*Note.* ^a^ not included in Study 2; ^b^ writing apprehension (KSAOs) and general media usage (outcomes) were included in both FtF and CM models. Parentheses show the results from the corresponding CM model; FtF = face-to-face, CM = computer-mediated, KSAOs = knowledge, skills, abilities, and other characteristics

## **Table 8**

*Sensitivity analysis for small df models. Robust fit-statistics for all SEM models (full sample; Study 2) and explained variance (R^2^)*

|  |  | **χ²** | **df** | **χ²- p value** | **CFI** | **RMSEA** | **SRMR** | **Model *R^2^* FtF/CM** |
| --- | --- | --- | --- | --- | --- | --- | --- | --- |
| SEM Model-fit | **Big Five generic as predictors** |  |  |  |  |  |  |  |
|  | Model for FtF/CM Motivation | 26.255 | 28 | .559 | 1.00 | .00 | .03 | .44/.02 |
|  | Model for FtF/CM Apprehension | 61.541 | 38 | .009 | .97 | .06 | .03 | .48/.23 |
|  | Model for FtF/CM Knowledge | 35.106 | 28 | .167 | .98 | .04 | .04 | .61/.30 |
|  | Model for FtF/CM Attentiveness** | 65.741 | 28 | <.001 | .89 | .09 | .05 | .35/.18 |
|  | Model for FtF/CM Expressiveness | 47.181 | 28 | .013 | .96 | .06 | .04 | .57/.24 |
|  | Model for FtF/CM Composure** | 82.526 | 28 | <.001 | .88 | .11 | .05 | .55/.28 |
|  | Model for FtF/CM Appropriateness | 39.884 | 28 | .068 | .97 | .05 | .05 | .40/.26 |
|  | Model for FtF/CM Effectiveness | 48.949 | 28 | .008 | .96 | .07 | .03 | .38/.22 |
|  | Model for FtF/CM Satisfaction | 33.013 | 28 | .235 | .99 | .03 | .02 | .55/.06 |
|  | Model for Media Usage | 43.169 | 25 | .013 | .95 | .07 | .04 | .15* |
| SEM Model-fit | **Big Five CM contextualized as predictors** |  |  |  |  |  |  |  |
|  | Model for FtF/CM Motivation | 29.017 | 28 | .412 | 1.00 | .01 | .03 | .08/.13 |
|  | Model for FtF/CM Apprehension | 69.148 | 38 | .001 | .97 | .07 | .03 | .13/.49 |
|  | Model for FtF/CM Knowledge | 59.929 | 28 | <.001 | .91 | .08 | .05 | .13/.48 |
|  | Model for FtF/CM Attentiveness | 58.092 | 28 | .001 | .92 | .08 | .05 | .08/.35 |
|  | Model for FtF/CM Expressiveness | 39.873 | 28 | .068 | .98 | .05 | .04 | .14/.58 |
|  | Model for FtF/CM Composure | 57.791 | 28 | .001 | .93 | .08 | .05 | .19/.58 |
|  | Model for FtF/CM Appropriateness | 54.003 | 28 | .002 | .93 | .08 | .05 | .33/.38 |
|  | Model for FtF/CM Effectiveness | 57.199 | 28 | .001 | .94 | .08 | .04 | .09/.29 |
|  | Model for FtF/CM Satisfaction | 19.342 | 28 | .887 | 1.00 | .00 | .02 | .16/.33 |
|  | Model for Media Usage | 30.389 | 25 | .210 | .99 | .04 | .03 | .20* |

*Note. ** We did not assess a FtF counterpart of media usage; ** the model did not exhibit satisfactory fit regarding all criteria. Therefore, misfit was

inspected and models modified until satisfactory fit was achieved. The prediction pattern remained similar compared to the unmodified models; FtF = face-to-face, CM = computer-mediated

## **Table 9**

*Sensitivity analysis for small df models. Robust fit-statistics for all SEM models (potential careless responders excluded; Study 2) and explained variance (R^2^)*

|  |  | **χ²** | **df** | **χ²- p value** | **CFI** | **RMSEA** | **SRMR** | **Model *R^2^* FtF/CM** |
| --- | --- | --- | --- | --- | --- | --- | --- | --- |
| SEM Model-fit | **Big Five generic as predictors** |  |  |  |  |  |  |  |
|  | Model for FtF/CM Motivation | 40.293 | 28 | .062 | 0.98 | .06 | .03 | .47/.04 |
|  | Model for FtF/CM Apprehension | 56.141 | 38 | .029 | .97 | .06 | .03 | .50/.15 |
|  | Model for FtF/CM Knowledge | 25.970 | 28 | 0.575 | 1.00 | .00 | .04 | .54/.42 |
|  | Model for FtF/CM Attentiveness** | 61.470 | 28 | <.001 | .87 | .10 | .06 | .34/.13 |
|  | Model for FtF/CM Expressiveness | 41.053 | 28 | .053 | .96 | .06 | .04 | .65/.24 |
|  | Model for FtF/CM Composure** | 65.932 | 28 | <.001 | .89 | .10 | .05 | .61/.21 |
|  | Model for FtF/CM Appropriateness | 53.349 | 28 | .003 | .91 | .09 | .05 | .30/.25 |
|  | Model for FtF/CM Effectiveness | 44.188 | 28 | .027 | .96 | .07 | .04 | .35/.20 |
|  | Model for FtF/CM Satisfaction | 31.858 | 28 | .280 | .99 | .03 | .03 | .55/.05 |
|  | Model for Media Usage | 31.762 | 25 | .165 | .97 | .05 | .04 | .20* |
| SEM Model-fit | **Big Five CM contextualized as predictors** |  |  |  |  |  |  |  |
|  | Model for FtF/CM Motivation | 28.286 | 28 | .449 | 1.00 | .01 | .03 | .06/.13 |
|  | Model for FtF/CM Apprehension | 59.601 | 38 | .014 | .97 | .07 | .03 | .08/.41 |
|  | Model for FtF/CM Knowledge | 45.764 | 28 | .018 | .93 | .07 | .05 | .07/.46 |
|  | Model for FtF/CM Attentiveness** | 56.310 | 28 | .001 | .89 | .09 | .06 | .08/.39 |
|  | Model for FtF/CM Expressiveness | 39.752 | 28 | .070 | .97 | .05 | .04 | .09/.56 |
|  | Model for FtF/CM Composure** | 64.064 | 28 | <.001 | .89 | .10 | .06 | .15/.56 |
|  | Model for FtF/CM Appropriateness** | 78.678 | 28 | <.001 | .86 | .12 | .07 | .24/.51 |
|  | Model for FtF/CM Effectiveness | 37.636 | 28 | .105 | .97 | .05 | .04 | .10/.33 |
|  | Model for FtF/CM Satisfaction | 30.416 | 28 | .344 | 1.00 | .03 | .03 | .14/.36 |
|  | Model for Media Usage | 33.147 | 25 | .127 | .97 | .05 | .05 | .17* |

*Note. ** We did not assess a FtF counterpart of media usage; ** the model did not exhibit satisfactory fit regarding all criteria. Therefore, misfit was

inspected and models modified until satisfactory fit was achieved. The prediction pattern remained similar compared to the unmodified models; FtF = face-to-face, CM = computer-mediated

## **Table 10**

*Univariate relative weight analyses: Individual results for multiple regression models (generic personality as predictor; full sample)*

| Univariate relative  weight analysis | |  |  | **Predictor variables - Big Five generic** | | | | |
| --- | --- | --- | --- | --- | --- | --- | --- | --- |
|  |  |  |  | **Relative weights** | | | | |
|  |  | *n* | Model *R^2^* | O | C | E | A | N |
| **KSAOs** |  |  |  |  |  |  |  |  |
| Motivation FtF | | 172 | 0.35 | 0.00 | 0.08 | 0.19 | 0.01 | 0.07 |
| Motivation CM | | 173 | 0.02 | 0.00 | 0.01 | 0.00 | 0.00 | 0.00 |
| Apprehension FtF | | 171 | 0.37 | 0.00 | 0.03 | 0.19 | 0.00 | 0.14 |
| Apprehension CM | | 171 | 0.19 | 0.01 | 0.01 | 0.06 | 0.01 | 0.11 |
| Knowledge FtF | | 172 | 0.41 | 0.00 | 0.06 | 0.20 | 0.01 | 0.14 |
| Knowledge CM | | 173 | 0.13 | 0.05 | 0.01 | 0.01 | 0.00 | 0.06 |
| Attentiveness FtF | | 172 | 0.22 | 0.01 | 0.08 | 0.11 | 0.01 | 0.02 |
| Attentiveness CM | | 173 | 0.12 | 0.02 | 0.02 | 0.07 | 0.00 | 0.01 |
| Expressiveness FtF | | 172 | 0.40 | 0.04 | 0.04 | 0.27 | 0.01 | 0.04 |
| Expressiveness CM | | 173 | 0.11 | 0.03 | 0.00 | 0.07 | 0.01 | 0.00 |
| Composure FtF | | 172 | 0.40 | 0.01 | 0.04 | 0.18 | 0.00 | 0.17 |
| Composure CM | | 173 | 0.16 | 0.04 | 0.01 | 0.03 | 0.03 | 0.06 |
|  |  |  |  |  |  |  |  |  |
|  |  | *n* | Model *R^2^* | O | C | E | A | N |
| **Outcomes** |  |  |  |  |  |  |  |  |
| Appropriateness FtF | | 172 | 22 | 0.01 | 0.09 | 0.01 | 0.10 | 0.01 |
| Appropriateness CM | | 173 | 17 | 0.00 | 0.06 | 0.00 | 0.10 | 0.00 |
| Effectiveness FtF | | 172 | 26 | 0.01 | 0.06 | 0.11 | 0.00 | 0.09 |
| Effectiveness CM | | 173 | 14 | 0.02 | 0.02 | 0.02 | 0.01 | 0.06 |
| Satisfaction FtF | | 172 | 42 | 0.00 | 0.08 | 0.20 | 0.01 | 0.13 |
| Satisfaction CM | | 173 | 4 | 0.00 | 0.01 | 0.00 | 0.00 | 0.02 |

*Note.* FtF = face-to-face, CM = computer-mediated, KSAOs = knowledge, skills, abilities, and other characteristics, O = Openness, C = Conscientiousness, E = Extraversion, A = Agreeableness, N = Neuroticism

## **Table 11**

*Univariate relative weight analyses: Individual results for multiple regression models (CM contextualized personality as predictor; full sample)*

| Univariate relative  weight analysis | |  |  | **Predictor variables - Big Five CM contextualized** | | | | |
| --- | --- | --- | --- | --- | --- | --- | --- | --- |
|  |  |  |  | **Relative weights** | | | | |
|  |  | *n* | Model *R^2^* | O | C | E | A | N |
| **KSAOs** |  |  |  |  |  |  |  |  |
| Motivation FtF | | 170 | 0.05 | 0.00 | 0.01 | 0.00 | 0.00 | 0.04 |
| Motivation CM | | 170 | 0.10 | 0.01 | 0.01 | 0.07 | 0.00 | 0.01 |
| Apprehension FtF | | 170 | 0.08 | 0.00 | 0.02 | 0.00 | 0.00 | 0.05 |
| Apprehension CM | | 170 | 0.37 | 0.01 | 0.03 | 0.12 | 0.00 | 0.21 |
| Knowledge FtF | | 170 | 0.07 | 0.00 | 0.01 | 0.00 | 0.01 | 0.05 |
| Knowledge CM | | 170 | 0.27 | 0.03 | 0.06 | 0.06 | 0.00 | 0.11 |
| Attentiveness FtF | | 170 | 0.04 | 0.00 | 0.01 | 0.01 | 0.00 | 0.01 |
| Attentiveness CM | | 170 | 0.21 | 0.05 | 0.04 | 0.12 | 0.01 | 0.00 |
| Expressiveness FtF | | 170 | 0.06 | 0.01 | 0.02 | 0.00 | 0.02 | 0.00 |
| Expressiveness CM | | 170 | 0.33 | 0.11 | 0.02 | 0.19 | 0.00 | 0.01 |
| Composure FtF | | 170 | 0.07 | 0.00 | 0.02 | 0.00 | 0.00 | 0.05 |
| Composure CM | | 170 | 0.30 | 0.06 | 0.06 | 0.05 | 0.02 | 0.10 |
|  |  |  |  |  |  |  |  |  |
|  |  | *n* | Model *R^2^* | O | C | E | A | N |
| **Outcomes** |  |  |  |  |  |  |  |  |
| Appropriateness FtF | | 170 | 0.18 | 0.00 | 0.03 | 0.01 | 0.10 | 0.04 |
| Appropriateness CM | | 170 | 0.23 | 0.00 | 0.03 | 0.03 | 0.17 | 0.01 |
| Effectiveness FtF | | 170 | 0.05 | 0.01 | 0.02 | 0.00 | 0.00 | 0.02 |
| Effectiveness CM | | 170 | 0.21 | 0.02 | 0.04 | 0.04 | 0.00 | 0.09 |
| Satisfaction FtF | | 170 | 0.09 | 0.01 | 0.02 | 0.00 | 0.00 | 0.06 |
| Satisfaction CM | | 170 | 0.24 | 0.04 | 0.03 | 0.05 | 0.01 | 0.10 |

*Note.* FtF = face-to-face, CM = computer-mediated, KSAOs = knowledge, skills, abilities, and other characteristics, O = Openness, C = Conscientiousness, E = Extraversion, A = Agreeableness, N = Neuroticism

## **Table 12**

*Summary of univariate relative weight analyses with personality (generic and CM framing) as predictor of KSAOs and Outcomes (full sample*)

|  |  | |  | | | **Study 2** | | | | | | | | | | | | |  |
| --- | --- | --- | --- | --- | --- | --- | --- | --- | --- | --- | --- | --- | --- | --- | --- | --- | --- | --- | --- |
|  |  | |  |  |  | **Big Five personality traits  (generic)** | | | | |  | | **Big Five personality traits  (CM framing)** | | | | | |  |
| **Model** | | **Results** | |  | O | | C | E | A | N | |  | | O | C | E | A | N | |
| ***FtF communication KSAOs*** | | Mean RW | |  | .01 | | .05 | .19 | .01 | .10 | |  | | .00 | .02 | .00 | .01 | .03 | |
|  | | Median RW | |  | .01 | | .05 | .19 | .01 | .11 | |  | | .00 | .02 | .00 | .00 | .04 | |
|  | | Range RW | |  | .00-.04 | | .03-.08 | .11-.27 | .00-.01 | .02-.17 | |  | | .00-.01 | .01-.02 | .00-.01 | .00-.02 | .00-.05 | |
| ***CM communication KSAOs*** | | Mean RW | |  | .02 | | .01 | .04 | .01 | .04 | |  | | .04 | .04 | .10 | .01 | .07 | |
|  | | Median RW | |  | .02 | | .01 | .04 | .00 | .03 | |  | | .04 | .03 | .09 | .00 | .06 | |
|  | | Range RW | |  | .00-.05 | | .00-.02 | .00-.07 | .00-.03 | .00-.11 | |  | | .01-.11 | .01-.06 | .05-.19 | .00-.02 | .00-.21 | |
| ***FtF communication outcomes*** | | Mean RW | |  | .01 | | .08 | .10 | .04 | .08 | |  | | .01 | .02 | .00 | .03 | .04 | |
|  | | Median RW | |  | .01 | | .08 | .11 | .01 | .09 | |  | | .01 | .02 | .00 | .00 | .04 | |
|  | | Range RW | |  | .00-.01 | | .06-.09 | .01-.20 | .00-.10 | .01-.13 | |  | | .00-.01 | .02-.03 | .00-.01 | .00-.10 | .02-.06 | |
| ***CM communication outcomes*** | | Mean RW | |  | .01 | | .03 | .01 | .04 | .03 | |  | | .02 | .04 | .04 | .06 | .07 | |
|  | | Median RW | |  | .00 | | .02 | .00 | .01 | .02 | |  | | .02 | .03 | .04 | .01 | .09 | |
|  | | Range RW | |  | .00-.02 | | .01-.06 | .00-.02 | .00-.10 | .00-.06 | |  | | .00-.04 | .03-.04 | .03-.05 | .00-.17 | .01-.10 | |

*Note:* RW are univariate relative weights; in Study 2, outcomes did not include attractiveness and clarity; due to missing data, *n* varies between *n* = 170

to 173 (Study 2) per analysis; FtF = face-to-face, CM = computer-mediated, KSAOs = knowledge, skills, abilities, and other characteristics, O =

Openness, C = Conscientiousness, E = Extraversion, A = Agreeableness, N = Neuroticism.

# **References**

Daly, J. A., & Miller, M. D. (1975). The empirical development of an instrument to measure writing apprehension. *Research in the Teaching of English*, *9*(3), 242-249.

McCroskey, J. C., Beatty, M. J., Kearney, P., & Plax, T. G. (1985). The content validity of the PRCA‐24 as a measure of communication apprehension across communication contexts. *Communication Quarterly*, *33*(3), 165-173. https://doi.org/10.1080/01463378509369595

R Core Team (2020). R: A language and environment for statistical computing [Computer software] (Version 3.6.3). Vienna, Austria: R Foundation for Statistical Computing. http://www.R-project.org/.

Schultze, M. (2017). stuart: Subtests using algorithmic rummaging techniques [R Package, Version 0.6.1]. Retrieved from https://bitbucket.org/martscht/stuart.

Schweizer, K. (2010). Some guidelines concerning the modeling of traits and abilities in test construction. *European Journal of Psychological Assessment*, *26*(1), 1-2.

http://doi.org/10.1027/1015-5759/a000001

Spitzberg, B. H. (2006). Preliminary development of a model and measure of computer‐mediated communication (CMC) competence*. Journal of Computer-Mediated Communication*, *11*(2), 629-666. https://doi.org/10.1111/j.1083-6101.2006.00030.x

Tonidandel, S., & LeBreton, J. M. (2015). RWA web: A free, comprehensive, web-based, and user-friendly tool for relative weight analyses. *Journal of Business and Psychology*, *30*(2), 207-216. https://doi.org/10.1007/s10869-014-9351-z

Tonidandel, S., LeBreton, J. M., & Johnson, J. W. (2009). Determining the statistical significance of relative weights. *Psychological Methods*, *14*(4), 387-399. https://doi.org/10.1037/a0017735

West, S. G., Taylor, A. B., & Wu, W. (2012). Model fit and model selection in structural equation modeling. In R. H. Hoyle (Ed.), *Handbook of structural equation modeling* (pp. 209–231). New York, NY: Guilford.
